# Doxorubicin-loading core-shell pectin nanocell: A novel nanovehicle for anticancer agent delivery with multidrug resistance reversal

**DOI:** 10.1371/journal.pone.0235090

**Published:** 2020-06-22

**Authors:** Jiabi Ouyang, Mohui Yang, Tian Gong, Jinlai Ou, Yani Tan, Zhen Zhang, Sha Li

**Affiliations:** 1 College of Pharmacy, Jinan University, Guangzhou, China; 2 International Cooperative Laboratory of Traditional Chinese Medicine Modernization and Innovative Drug Development of Chinese Ministry of Education, College of Pharmacy, Jinan University, Guangzhou, China; 3 Guangdong Province Key Laboratory of Pharmacodynamic Constituents of TCM and New Drugs Research, Guangzhou, China; St. John's University, UNITED STATES

## Abstract

Tumor is a prevalent great threat to public health worldwide and multidrug resistance (MDR) of tumor is a leading cause of chemotherapy failure. Nanomedicine has shown prospects in overcoming the problem. Doxorubicin (DOX), a broad-spectrum anticancer drug, showed limited efficacy due to MDR. Herein, a doxorubicin containing pectin nanocell (DOX-PEC-NC) of core-shell structure, a pectin nanoparticle encapsulated with liposome-like membrane was developed. The DOX-PEC-NC, spheroid in shape and sized around 150 nm, exerted better sustained release behavior than doxorubicin loading pectin nanoparticle (DOX-PEC-NP) or liposome (DOX-LIP). *In vitro* anticancer study showed marked accumulation of doxorubicin in different tumor cells as well as reversal of MDR in HepG2/ADR cells and MCF-7/ADR cells caused by treatment of DOX-PEC-NC. In H_22_ tumor-bearing mice, DOX-PEC-NC showed higher anticancer efficacy and lower toxicity than doxorubicin. DOX-PEC-NC can improve anticancer activity and reduce side effect of doxorubicin due to increased intracellular accumulation and reversal of MDR in tumor cells, which may be a promising nanoscale drug delivery vehicle for chemotherapeutic agents.

## Introduction

Cancer is the leading cause of death and has become a serious public health problem worldwide [1,2]. Cytotoxicity-based chemotherapy is one of the most commonly used means of cancer treatment [3], however, the efficacy of chemotherapy is seriously weakened because of the poor tumor targeting of chemotherapeutic agents and acquired chemotherapy resistance of tumors [4,5]. Multidrug resistance (MDR), which is cross-resistance to anticancer drugs with different structures or mechanisms, reduces the accumulation of chemotherapeutic agents in tumor cells and leads to the failure of chemotherapy [6]. It has been reported that chemoresistance mechanisms involved drug efflux mediated by the up-regulation of ATP binding cassette (ABC) transporters, the most common of which is ABCB1-encoded P-glyoprotein (P-gp) [7,8]. The overexpressed P-gp protein on tumor cell membrane pumps the chemotherapeutic agents out of cells, resulting in higher drug dose required to achieve an effective therapeutic concentration [9], which brings increased toxic and side effects together without surprise. Doxorubicin (DOX) is such a typical first-line anthracycline chemotherapeutic agent, of good anticancer effect accompanied with troublesome toxic side effects such as chronic cardiotoxicity [10], hepatotoxicity [11], nephrotoxicity [12], testicular damage [13] and multidrug resistance [14], which make it a double-edged sword and severely impede its clinical application.

To ensure expected therapeutic efficacy as well as lower down the toxic and side effect, microparticle drug delivery systems, especially in nanometer size, were explored to enable targeting delivery of chemotherapeutic agents in treatment of solid tumors. Nano-size drug delivery systems, including liposomes, polymeric micelles and nanoparticles, have been widely used to deliver DOX and other chemotherapeutic agents [15–18]. Different kinds of materials were used to develop nanoscale delivery vehicles of DOX to achieve acceptable anticancer effect at relatively low dose while reduce toxic and side effects [19]. The nanoscale vehicles were generally inclined to accumulate in tumor tissue through various targeting actions and enhanced permeability and retention (EPR) effect, thus to release the loading drug slowly at the tumor site and exert good anticancer efficacy with low toxic side effects [20,21].

Pectin is a non-toxic, biocompatible and biodegradable anionic polysaccharide [22,23], which can be obtained from plants at a low cost [24]. Pectin nanoparticle has been receiving great attention in cancer treatment due to its superior features, such as biocompatibility, good drug loading capacity, sustained drug release and targeted localization [25]. However, the numerous hydroxyl groups in the structure cause the pectin nanoparticle to swell easily in an aqueous environment [26], which may weaken its sustained release action. If pectin is wrapped within a hydrophobic material membrane to limit its swelling, the problem is possible to be overcome. Liposome is a spherical vesicle with an aqueous core surrounded by phospholipid bilayer membrane, which can entrap both water-soluble and liposoluble drugs. The cell membrane-like structure makes it of good biocompatibility and efficient delivery ability, which makes it a widely used attractive drug delivery nanocarrier [27–29]. On the other hand, liposome has disadvantages like particle aggregation, undesired membrane fusion, phospholipids degradation and drug leakage during storage [30,31]. Nanocell was first termed in research of Shiladitya Sengupta *et al* [32], which was a kind of nanoscale delivery system featured with a PLGA nanoparticle entrapped in an pegylated-lipid envelop in that work. It intended to develop a model of ‘integrative’ approach in cancer therapy by loading both combretastatin-A4 and DOX to achieve expected anticancer effect from anti-angiogenesis and chemotherapy at the same time. There have been some studies on nanocells with nanoparticle core prepared with metal or other synthetic polymer materials, like poly(acrylic acid) [33], polystyrene [34], magnetic/plasmonic materials [35], polyelectrolyte capsule [36]. However, the possible unpredictable toxicity might be an obsession in future application. Although polysaccharide materials of good biocompatibility and safety like pectin were commonly used in preparing nanoscale drug delivery systems, not so much has been investigated in polysaccharide nanocells developed with nanoparticle core of polysaccharide materials so far. Herein, a novel nanocell was developed by encapsulating pectin nanoparticle core in liposome-like membrane to further discuss the advantages of combining technology of nanoparticle and liposome in both pharmaceutical characteristics and application in anticancer therapy.

**Fig 1:**
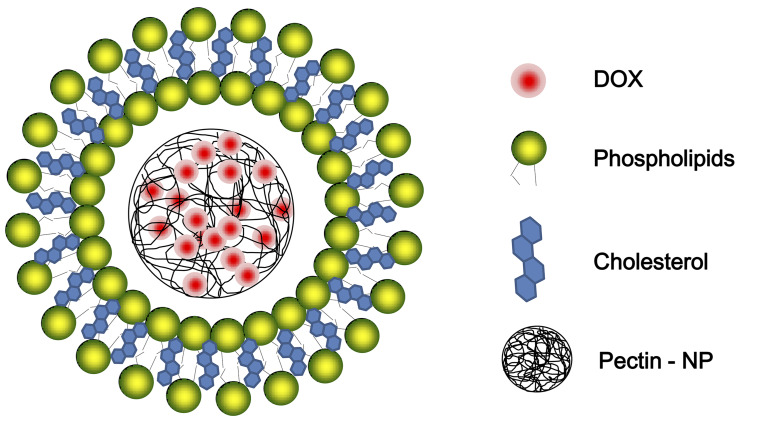
Schematic illustration of DOX-PEC-NC.

In this work, doxorubicin was used as model chemotherapeutic agent to attempt development of a novel doxorubicin-containing pectin nanocell (DOX-PEC-NC) of core-shell structure, a pectin nanoparticle encapsulated with liposome-like membrane (Fig 1). The features of DOX-PEC-NC were characterized and its anticancer activity *in vitro* and *in vivo* was investigated as well, including MDR reversal action.

## Materials and methods

### Materials

Doxorubicin hydrochloride (DOX) was received from Hisun Pharmaceutical Co., Ltd. (Zhejiang,China). Low-methoxylated esterified amidated pectin was obtained from CPKelco Company (USA). Oleic acid and cholesterol (CHOL) was purchased from Aladdin Industrial Corporation (Shanghai, China). Sephadex G-50 was provided by Pharmacia Industrial Corporation (USA). Soy lecithin was obtained from Lipoid Industrial Corporation (Germany). 3-(4,5-dimethyl-thiazol-2-yl)-2,5-diphenyl tetrazolium bromide (MTT), sodium bis(2-ethylhexyl) sulfosuccinate (AOT) and dimethyl sulfoxide (DMSO) were received from Sigma-Aldrich Chemical Co., Ltd. (St. Louis, MO, USA). Fetal bovine serum (FBS) and RPMI-1640 media were provided by Gibco (USA). All other solvents and reagents were analytical grade.

### Cell culture

The human cervix adenocarcinoma cell line Hela, the human hepatocellular carcinoma cell line HepG2, the human breast adenocarcinoma cell line MCF-7, the human breast adenocarcinoma cell line MDA-MB-231, the human lung carcinoma cell line A549, the human lung carcinoma cell line NCI-H1299 were obtained from ATCC, the doxorubicin (ADR)-resistant human hepatocellular carcinoma cell line HepG2/ADR and human breast adenocarcinoma cell line MCF-7/ADR were obtained from Shanghai Aiyan Biological Technology Co., LTD. (Shanghai, China) and Nanjing KeyGen Biotech. Co. Ltd. (Nanjing, China), respectively. These cell lines were authenticated as having no cross-contamination of other human cell lines using the STR Multi-Amplification Kit (Microreader 21 ID System) and were tested negative for mycoplasma using the Mycoplasma Detection Set (M&C Gene Technology). The cells were cultured in RPMI-1640 medium supplemented with 10% FBS, 100 IU/mL of penicillin and streptomycin in an incubator at 37°C under 5% CO_2_ atmosphere. In order to maintain a resistant phenotype, HepG2/ADR and MCF-7/ADR cells were cultured in a medium containing DOX, and the DOX concentration in the medium were 1000 ng/mL and 400 ng/mL, respectively, and then cultured for one week with DOX-free medium before experiment [37].

The mouse hepatocarcinoma cell line H_22_ was obtained from Shanghai Huzheng Biological Technology Co., Ltd. (Shanghai, China). 0.2 mL H_22_ cell suspension (1 × 10^7^/mL) was inoculated intraperitoneally in male Kunming mice to cause ascites, and tumor cells in the ascites were extracted for subsequent establishment of H_22_ tumor bearing mice model.

### Animals

Male Kunming mice were provided by Guangdong Medical Laboratory Animal Center (Guangdong, China). The animals were housed in an environmentally controlled breeding room with free access to standard laboratory food and water. All animals were kept 7 days for acclimation before experiments.

### Ethics statement

All animal experiments described in the study were approved by Laboratory Animal Ethics Committee of Jinan University and conducted in accordance with the guidelines of Laboratory Animal Ethics Committee of Jinan University. At the end of the experiment, the mice were anesthetized with pentobarbital sodium before sacrificed by cervical dislocation. Animals were monitored and properly handled throughout the experiment, and every effort was made to minimize suffering or pain.

### Preparation and characterization of DOX-PEC-NC

Doxorubicin loading pectin nanoparticle (DOX-PEC-NP) was firstly prepared as previously reported [38,39]. The nanoparticle was enveloped with liposome-like membrane by reverse phase evaporation method to prepare doxorubicin loading pectin nanocell (DOX-PEC-NC), and pH gradient process was combined together to increase doxorubicin loading rate. Briefly, soybean lecithin and cholesterol was dissolved in a mixture solvent of chloroform and n-hexane to prepare organic phase. Acidic aqueous suspension of DOX-PEC-NP as internal aqueous phase was then dispersed in organic phase by ultrasonication to prepare a stable W/O type emulsion. The emulsion was evaporated under reduced pressure to remove organic solvent followed by addition of an aliquot of distilled water containing doxorubicin as external water phase to hydrate the forming gel. The suspension was treated with ultrosonication to obtain DOX-PEC-NC. Doxorubicin loading liposome (DOX-LIP) was prepared as the same method described above by not adding DOX-PEC-NP in the internal aqueous phase. The prepared DOX-PEC-NC was characterized by morphology, size distribution, ζ potential, entrapment efficiency (EE%) and drug loading rate (DL%). The DOX-PEC-NC was also analyzed by Fourier transform infrared spectrometer (FTIR; EQUINOX 55, Germany) to attempt to understand its structure. The appearance was observed under transmission electron microscope (TEM; TECNAI 10, Netherlands). The particle size, polydispersity index (PDI) and ζ potential were measured using Zetasizer Nano ZS (Malvern, UK). The free doxorubicin and DOX-PEC-NC was separated using Sephadex G-50 column, and drug concentration was measured using high performance liquid chromatography (HPLC) to calculate EE% and DL% of DOX-PEC-NC as follows.

$$EE\%=\frac{W_{wrapped}}{W_{total}}\times 100\%\;DL\%=\frac{W_{wrapped}}{\left(W_{wrapped}+W_{vehicle}\right)}\times 100\%$$

W_wrapped_, W_total_ and W_vehicle_ in the equation represent the weight of drug wrapped in drug delivery vehicle, the total weight of drug used and the weight of drug delivery vehicle in the tested sample, respectively.

### *In vitro* drug release test

The *in vitro* release behavior of DOX-PEC-NC was investigated by dialysis bag method [40] in normal saline and phosphate buffer solution (PBS, pH 6.8 and 7.4), respectively. Briefly, DOX-PEC-NC was dispersed in a small volume of release medium and added into dialysis bag, and then the bag was placed in an aliquot of release medium to carry out drug release test at 37°C under 100 rpm of stirring. The samples were collected at 0.5, 1.0, 1.5, 2.0, 4.0, 8.0, 12.0, 24.0, 36.0, 48.0 and 72.0 h followed by supplement of fresh medium. The concentration of samples was analyzed using HPLC, and the release curve was plotted with accumulative drug release percentage versus time.

### *In vitro* cytotoxicity assay

The MTT staining assay was adopted to investigate the cytotoxicity of DOX-PEC-NC in sensitive tumor cell lines (Hela, HepG2, MCF-7, MDA-MB-231, A549 and NCI-H1299 cells) and drug resistant tumor cell lines (MCF-7/ADR and HepG2/ADR cells), respectively. Briefly, 3 × 10^3^ cells in logarithmic growth stage were inoculated in each well of 96-well plate and cultured overnight (37°C, 5% CO_2_), and then incubated with DOX-PEC-NC for 48 and 72 hours in sensitive tumor cell lines and 72 hours in drug resistant tumor cell lines, respectively. The concentration of doxorubicin ranged from 0.25 to 4.0 μg/mL in Hela, HepG2, MCF-7, MDA-MB-231, A549 and NCI-H1299 cells, while 3.125 to 100 μg/mL in MCF-7/ADR and HepG2/ADR cells. After incubation with DOX-PEC-NC, 15 μL of MTT working solution (5 mg/mL) was added in each well and the cells were incubated for another 4 hours. Then the supernatant was replaced by DMSO of 150 μL/well, and the absorbance at 570 nm was measured with microplate reader (Bio-Tek, USA) to calculate survival rate of cells and half-maximal inhibitory concentration (IC_50_) in experimental groups. The reversal fold of drug resistance was calculated by dividing IC_50_ value of DOX by that of DOX-NP, DOX-LIP or DOX-PEC-NC in MCF-7/ADR and HepG2/ADR cells, respectively. Cells without treatment were set as normal control group, blank liposome (LIP) and PEC-NC group was set to figure out the toxicity of these nanoscale drug delivery vehicles in cells, and doxorubicin and DOX-LIP group was set for comparison as well.

### Intracellular uptake of doxorubicin

The cells were seeded in six-well plate with a density of 2×10^5^ cells per well and cultured (37°C, 5% CO_2_) for 24 hours. The Hela, HepG2, MCF-7, MDA-MB-231, A549 and NCI-H1299 cells were incubated with DOX-PEC-NC at doxorubicin concentration of 1 μg/mL for 1 hour while MCF-7/ADR cells were at 5, 10 and 20 μg/mL for 4 hours, respectively. After incubation, the cells were washed three times with cold PBS, digested into cell suspension and dispersed in cold PBS, and then detected by flow cytometry (ACEA Biosciences, USA). Also, the intracellular uptake of doxorubicin in HepG2, MCF-7, HepG2/ADR and MCF-7/ADR cells was directly observed under inverted fluorescence microscopy. The HepG2 and MCF-7 cells were incubated with DOX-PEC-NC at doxorubicin concentration of 5 μg/mL for 2 and 6 hours, and HepG2/ADR and MCF-7/ADR cells at 20 μg/mL for 6 hours. After treatment, the medium was discarded and the cells were washed 3 times with cold PBS. A certain amount of 4% paraformaldehyde was added, and the cells were fixed for 30 minutes and then observed and photographed under an inverted fluorescence microscope (Carl Zeiss, Germany). Cells without treatment were set as normal control group, and doxorubicin group was set for comparison as well.

### Intracellular absorption and retention of doxorubicin

To determine the absorption of doxorubicin, cells in logarithmic growth stage were inoculated in 96-well plate with a density of 3 × 10^3^ cells per well and cultured overnight (37°C, 5% CO_2_), and then incubated with DOX-PEC-NC. The concentration of doxorubicin was 10 μg/mL in HepG2 and MCF-7 cells, and 100 μg/mL in MCF-7/ADR and HepG2/ADR cells. After 1, 2, 3, 4, and 5 hours of incubation under same condition, the cells were washed three times with cold PBS, then 100 μL of RIPA lysis buffer was added, and the fluorescence intensity (λexcitation = 485 nm, λemmision = 590 nm) was measured by a microplate reader after 30-minute shaking. To determine the retention of intracellular doxorubicin, HepG2, MCF-7, HepG2/ADR and MCF-7/ADR cells were incubated with DOX-PEC-NC under same condition described above for 4 hours, respectively. After washing three times with cold PBS, fresh drug-free medium was added to incubate for 0.5, 1.0, 2.0, and 4.0 hours, respectively. The cells were then washed three times with cold PBS, treated with 100 μL of RIPA lysis buffer under shaking for 30 minutes, and the fluorescence intensity was measured. The fluorescence intensity of intracellular doxorubicin at t = 0 was taken as 100% to calculate the retention percentage of doxorubicin with time. Cells without treatment were set as normal control group, and doxorubicin group was set for comparison as well.

### *In vivo* anticancer efficacy study

In order to evaluate the therapeutic effect, 1 × 10^7^ H_22_ cells were subcutaneously injected into the right front armpit of male Kunming mice (20 ± 2 g) to produce a tumor-bearing mice model. The successfully modeled mice with tumor volume not less than 100 mm^3^ were randomly divided into 9 groups, each group 10 mice. The first day of treatment was set as Day 0. The mice were injected with water for injection (model group), doxorubicin solution (5.0 mg/kg), DOX-LIP (5.0 mg/kg), DOX-PEC-NP (5.0, 2.5 and 1.0 mg/kg) and DOX-PEC-NC (5.0, 2.5 and 1.0 mg/kg) once a day via tail vein on Day 0, 4 and 8, respectively. The body weight and tumor volume of the mice were measured daily, and the general status was observed as well. Tumor volume was calculated using the formula (a × b^2^/2), where a and b denote the longest and shortest dimensions of the tumor tissues, respectively. All the mice were sacrificed on day 10, the tissues of tumor, thymus and spleen were collected and weighed, and thymus index or spleen index was calculated by equation of Thymus (Spleen) index = Weight of thymus (spleen) (mg)/ Weight of mouse (g). The inhibitory rate of tumor growth was calculated by the following equation using the mean weight of tumor tissues of model group as 100%, where ${\bar{W}}_{test\;group}$ and ${\bar{W}}_{model\;group}$ was the mean weight of tumor tissues of test and model group, respectively.

$$IR\%=\left(1-\frac{{\bar{W}}_{test\;group}}{{\bar{W}}_{model\;group}}\right)\times 100\%$$

### Statistical analysis

All data were expressed as mean ± standard deviation (SD). Statistical analysis was performed using SPSS software 19.0. The significant difference was analyzed using Student's t-test and *P <* 0.05 was considered statistically significant.

## Results and discussion

### Preparation and characterization of DOX-PEC-NC

**Fig 2:**
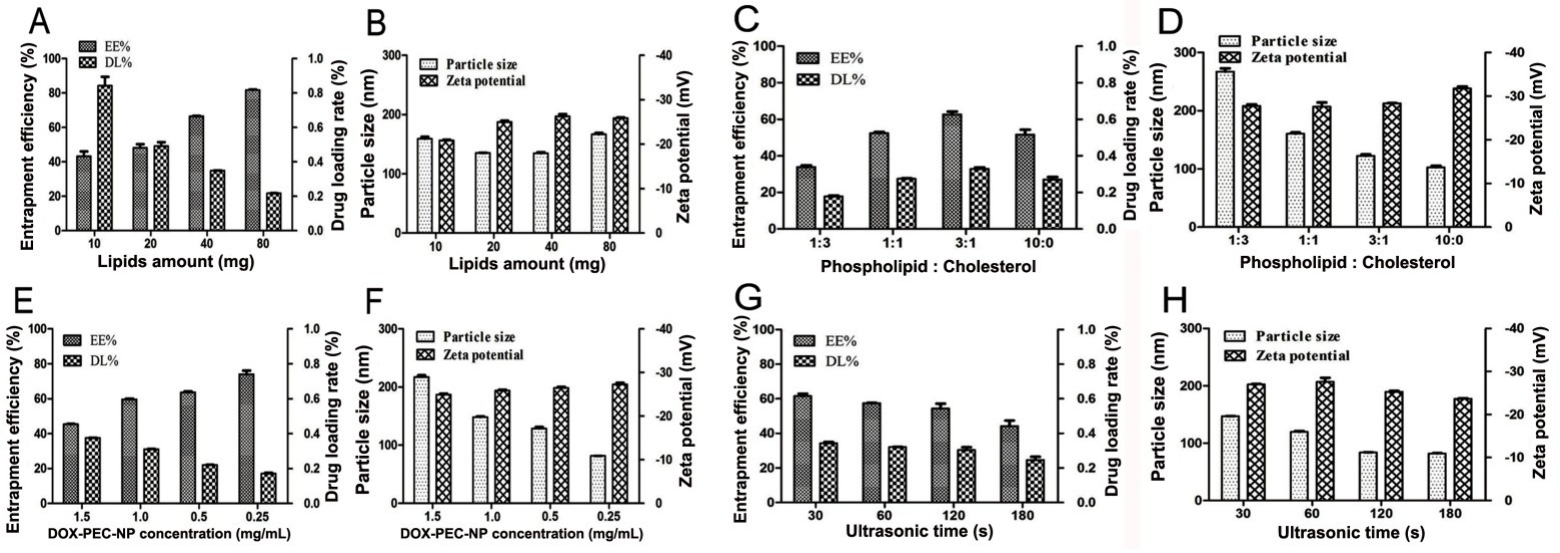
Influence of total amount of lipid (A, B), ratio of phospholipid to cholesterol (C, D), DOX-PEC-NP concentration in internal aqueous phase (E, F) and ultrasonic time of dispersion after hydration of lipid gel (G, H) on EE%, DL%, particle size and zeta potential of DOX-PEC-NC (n = 3).

Different factors were investigated to optimize the formulation and process of DOX-PEC-NC, including the total amount of lipid, ratio of lecithin to cholesterol, DOX-PEC-NP concentration in aqueous suspension, ultrasonic time of dispersion after hydration of lipid gel, and content of doxorubicin in the external aqueous phase. The results (Fig 2 and Table 1) showed that all of the investigated factors affected zeta potential of DOX-PEC-NC slightly, and the potential was around -20 to -30 mV. The particle size of DOX-PEC-NC was influenced obviously by the ratio of phospholipid to cholesterol (Fig 2D), concentration of DOX-PEC-NP in aqueous suspension (Fig 2F) and time of ultrasonic dispersion (Fig 2H).

**Table 1:** The influence of doxorubicin content added in the external aqueous phase on features of DOX-PEC-NC ($\bar{X}\pm SD$, n = 3).

Doxorubicin (mg)	Particle size (nm)	PDI	zeta potential (mV)	EE%	DL%
0	162.93 ± 1.80	0.252 ± 0.019	-24.37 ± 0.71	47.75 ± 0.69	0.87 ± 0.03
0.5	156.67 ± 1.91	0.245 ± 0.016	-22.87 ± 0.77	43.41 ± 0.65	1.12 ± 0.02
1.0	152.27 ± 2.04	0.246 ± 0.011	-22.20 ± 1.05	41.20 ± 0.40	1.84 ± 0.02
1.5	151.10 ± 1.91	0.230 ± 0.017	-19.63 ± 0.59	37.09 ± 0.45	2.05 ± 0.04

**Fig 3:**
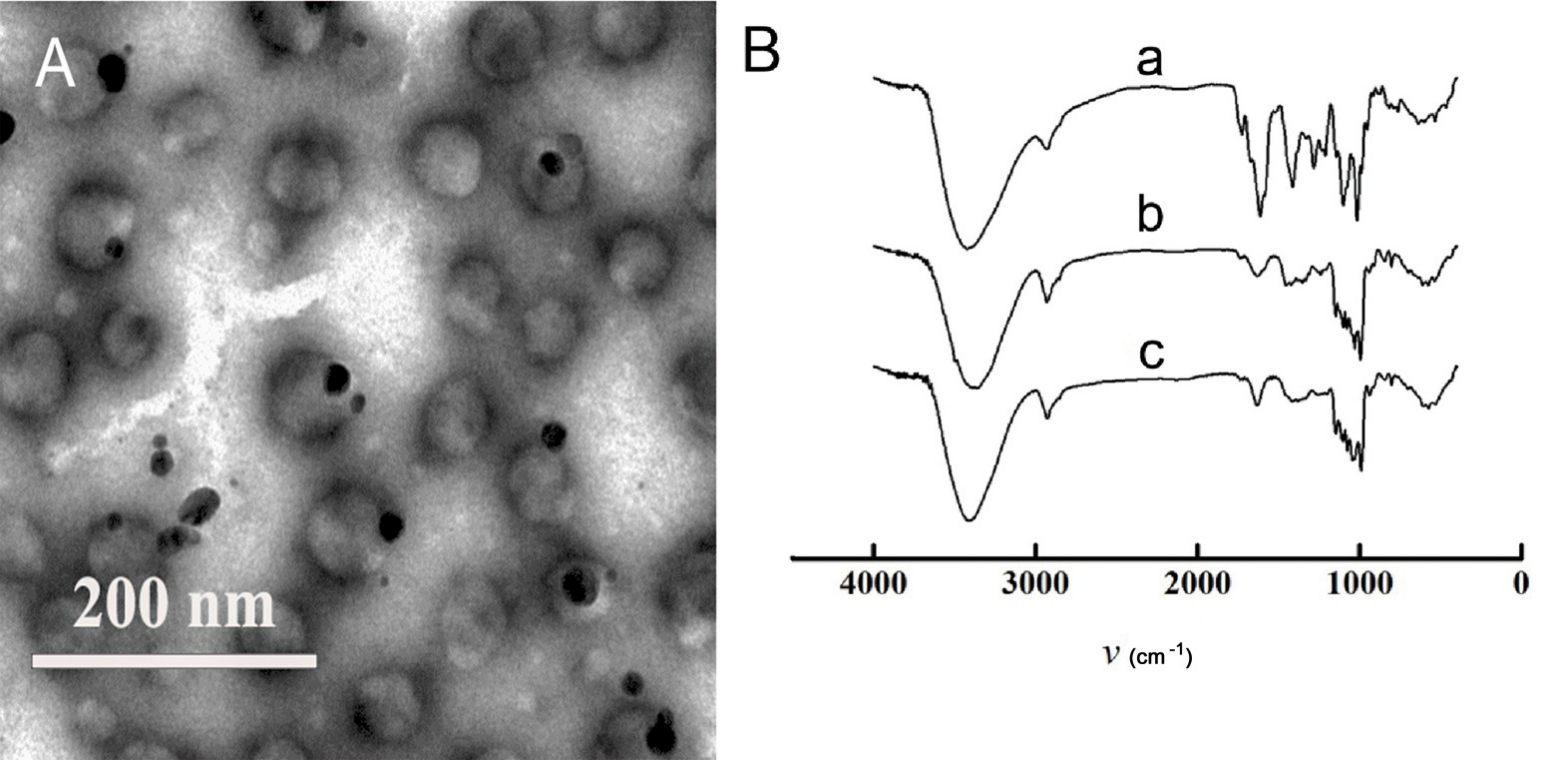
Morphology of DOX-PEC-NC observed under TEM (A) and FTIR spectra of DOX-PEC-NC (B) (a—DOX-PEC-NP, b—DOX-LIP, c—DOX-PEC-NC).

Except ultrasonic time, the other four factors showed significant effect on the drug loading capacity of DOX-PEC-NC (*P* < 0.05 or *P* < 0.01). As shown in Fig 2A, with the increase of total amount of lecithin and cholesterol, the EE% of DOX-PEC-NC increased gradually, and the DL% decreased markedly. As the ratio of lecithin to cholesterol increased, the EE% and DL% increased first and then decreased (Fig 2C). The increase of DOX-PEC-NP concentration enhanced the DL% but reduced the EE% of DOX-PEC-NC (Fig 2E). To further improve the drug loading capacity, ammonium sulfate solution or acidic water solution was used to prepare DOX-PEC-NP aqueous suspension to make an ammonium sulfate or pH gradient between internal and external aqueous phase. The use of ammonium sulfate solution in internal aqueous phase caused structural damage of DOX-PEC-NP, while dilute sulfuric acid or acidic PBS made the particle size of DOX-PEC-NC exceed 3000 nm. When DOX-PEC-NP dispersed in dilute hydrochloric acid solution, DOX-PEC-NC with higher DL% was successfully prepared by adding a proper amount of doxorubicin in external aqueous phase. The content of doxorubicin in external aqueous phase had slight influence on EE% but great influence on DL% of DOX-PEC-NC (Table 1). Finally, a negatively charged spheroid nanoscale DOX-PEC-NC around 150 nm was produced through optimization, the size distribution was narrow and DL% was around 1.8% (Fig 3A, Table 1). The particle size observed under TEM was smaller than hydrodynamic particle size due to the shrinkage after losing water [41].

The infrared spectra of DOX-PEC-NP, DOX-LIP and DOX-PEC-NC (Fig 3B) were compared and analyzed to discuss the structure of DOX-PEC-NC. The spectrum of DOX-PEC-NP showed main absorption peaks at 3407, 2926, 1743, 1618, 1423, 1331, 1235, 1144, 1103 cm^-1^ and DOX-LIP at 3362, 2930, 1636, 1454, 1240, 1149, 1101 cm^-1^. The main absorption peaks of DOX-PEC-NC were extremely close to those of DOX-LIP, however, the absorption peaks of DOX-PEC-NP at 1743, 1618 and 1423 cm^-1^ weakened or disappeared in the spectrum of DOX-PEC-NC. The results may indicate that DOX-PEC-NC has the designed core-shell structure with DOX-PEC-NP entrapped in liposome-like membrane. It led to failure to fully detect the group structure of entrapped DOX-PEC-NP but the group structure of liposome-like membrane and DOX loaded by pH gradient process was showed chiefly.

Also, we attempted to increase the storage stability of DOX-PEC-NC by lyophilization. When D-trehalose was used as lyoprotectant, the loose freeze-dried powder of DOX-PEC-NC was obtained. The dried DOX-PEC-NC powder was able to re-dispersed rapidly in water, showed spheroid morphology of similar size as that before freeze-drying. The lyophilization had little influence on the encapsulation rate and drug loading rate. Under storage in closed vials without sealing at 4°C, the lyophilized DOX-PEC-NC remained stable in morphology, size distribution and drug loading rate at least within 2 months.

### *In vitro* release behavior of DOX-PEC-NC

**Fig 4:**
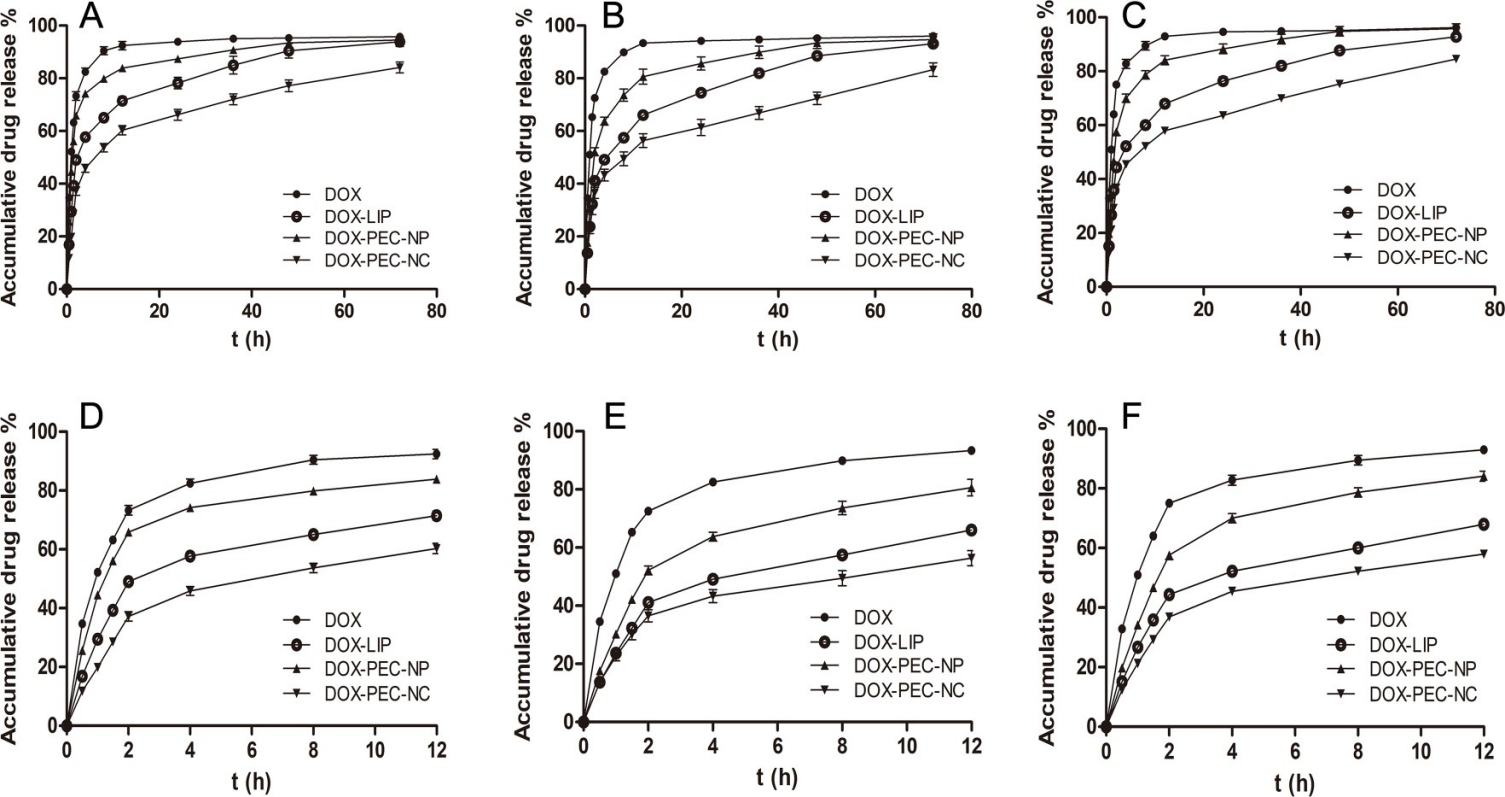
Drug release profiles of DOX, DOX-LIP, DOX-PEC-NP and DOX-PEC-NC in normal saline (A, D), PBS (pH 6.8; B, E) and PBS (pH 7.4; C, F) for 72 hours and 12 hours (n  =  3).

The drug release behavior of DOX-PEC-NP, DOX-LIP and DOX-PEC-NC was compared in three different mediums, normal saline, PBS (pH 7.4) to simulate the normal physiological condition and PBS (pH 6.8) to simulate the microenvironment of tumor. Compared with doxorubicin powder, DOX-PEC-NP, DOX-LIP and DOX-PEC-NC released drug slowly to different extent (Fig 4). In the tested three mediums, DOX-PEC-NP showed the poorest sustained release behavior with great burst effect, more than 50% to even about 65% of drug released at 2 hour and 80–84% released at 12 hour. DOX-LIP exerted better sustained release behavior than DOX-PEC-NP with some burst effect, more than 40% to about 49% of drug released at 2 hour and 82–85% released at 36 hour. The surface adsorbed drug on DOX-PEC-NP caused the burst effect, and the easy swelling and ion exchange of PEC-NP in medium made its sustained drug release behavior not as good as DOX-LIP, in which the drug passed through the lipid membrane to release. The drug release from DOX-PEC-NC was the slowest without obvious burst effect, less than 40% of drug released at 2 hour and around 84% released at 72 hour. It further confirmed the core-shell structure of DOX-PEC-NC from another aspect. In DOX-PEC-NC, the drug should release first from DOX-PEC-NP before passing through the lipid membrane to enter medium, and the membrane limited the swell and ion exchange of PEC-NP, which made drug release even slower than DOX-LIP.

The release data of DOX-PEC-NC were fitted with zero-order kinetic, first-order kinetic, two-phase kinetic and Higuchi equation using SPSS software 19.0, and the results were judged by the goodness of fit (r) (Table 2). The results showed that two-phase kinetic equation fitted the release data of DOX-PEC-NC well with the highest r value more than 0.99. It indicated that drug release from DOX-PEC-NC followed two-phase dynamic process. The release behavior of DOX-PEC-NP and DOX-LIP showed same pattern.

**Table 2:** The model fitting of drug release behavior of DOX-PEC-NC, DOX-PEC-NP and DOX-PEC-LIP.

Model	Goodness of fit (r)								
	DOX-PEC-NP			DOX-LIP			DOX-PEC-NC		
	NS	PBS		NS	PBS		NS	PBS	
		pH 6.8	pH 7.4		pH 6.8	pH 7.4		pH 6.8	pH 7.4
Zero order	0.6564	0.7370	0.7029	0.7865	0.8338	0.8126	0.8234	0.8434	0.8350
First order	0.8757	0.9180	0.9059	0.9535	0.9699	0.9602	0.9359	0.9481	0.9463
Higuchi	0.8099	0.8786	0.8519	0.9108	0.9436	0.9293	0.9352	0.9438	0.9402
Two-phase	0.9993	0.9973	0.9982	0.9976	0.9987	0.9986	0.9971	0.9947	0.9989

### Anticancer effect of DOX-PEC-NC *in vitro*

#### Cytotoxicity of DOX-PEC-NC in different tumor cell lines

**Fig 5:**
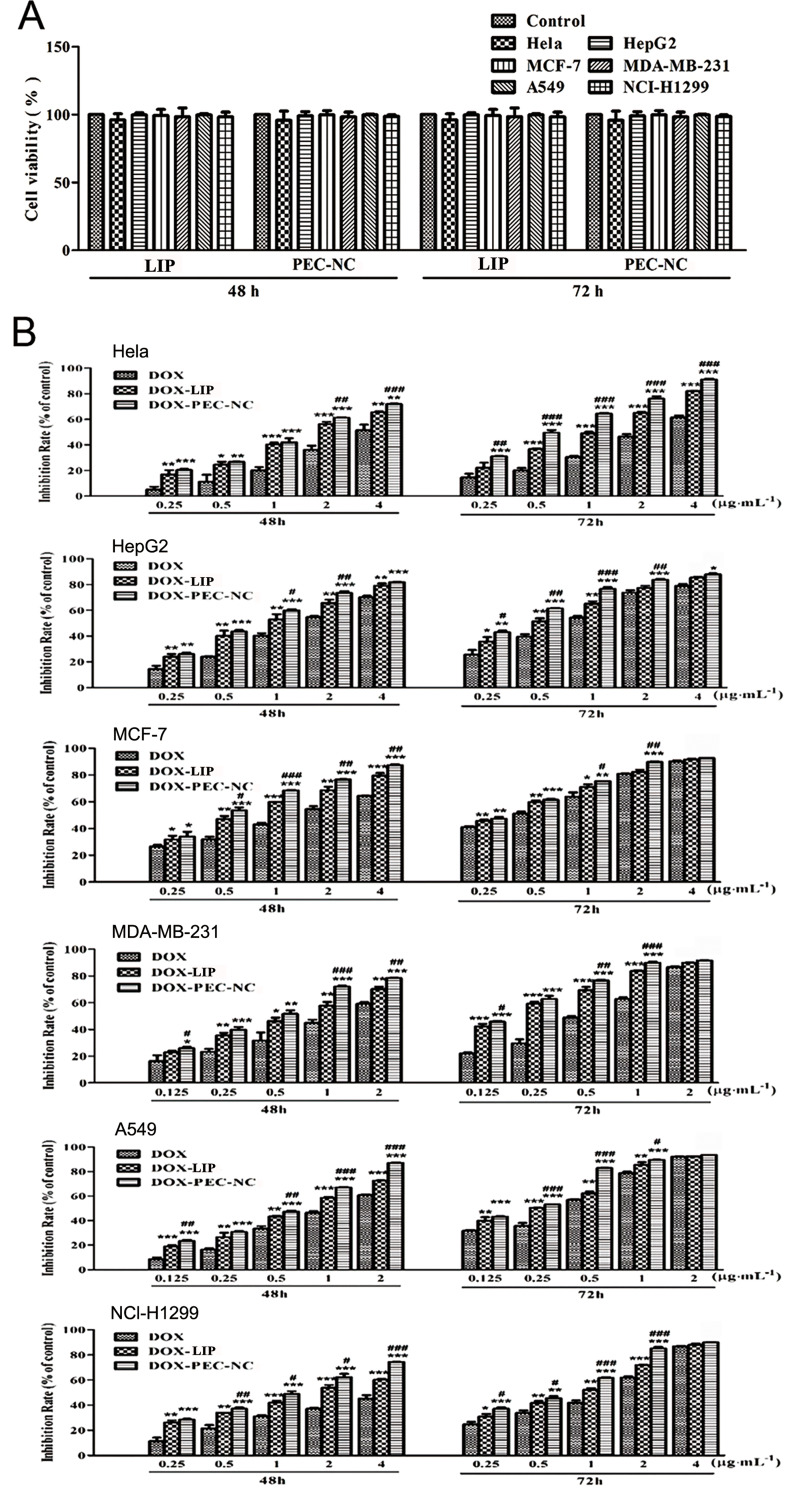
The effect of PEC-NC (A) and DOX-PEC-NC (B) on cell viability and inhibition rate in Hela, HepG2, MCF-7, MDA-MB-231, A549 and NCI-H1299 cells after 48- and 72-hour treatment at different drug concentration of doxorubicin (n = 3; *P < 0.05, **P < 0.01 and ***P < 0.001, compared with DOX; ^#^*P* < 0.05, ^##^*P* < 0.01 and ^###^*P* < 0.001, compared with DOX-LIP).

**Fig 6:**
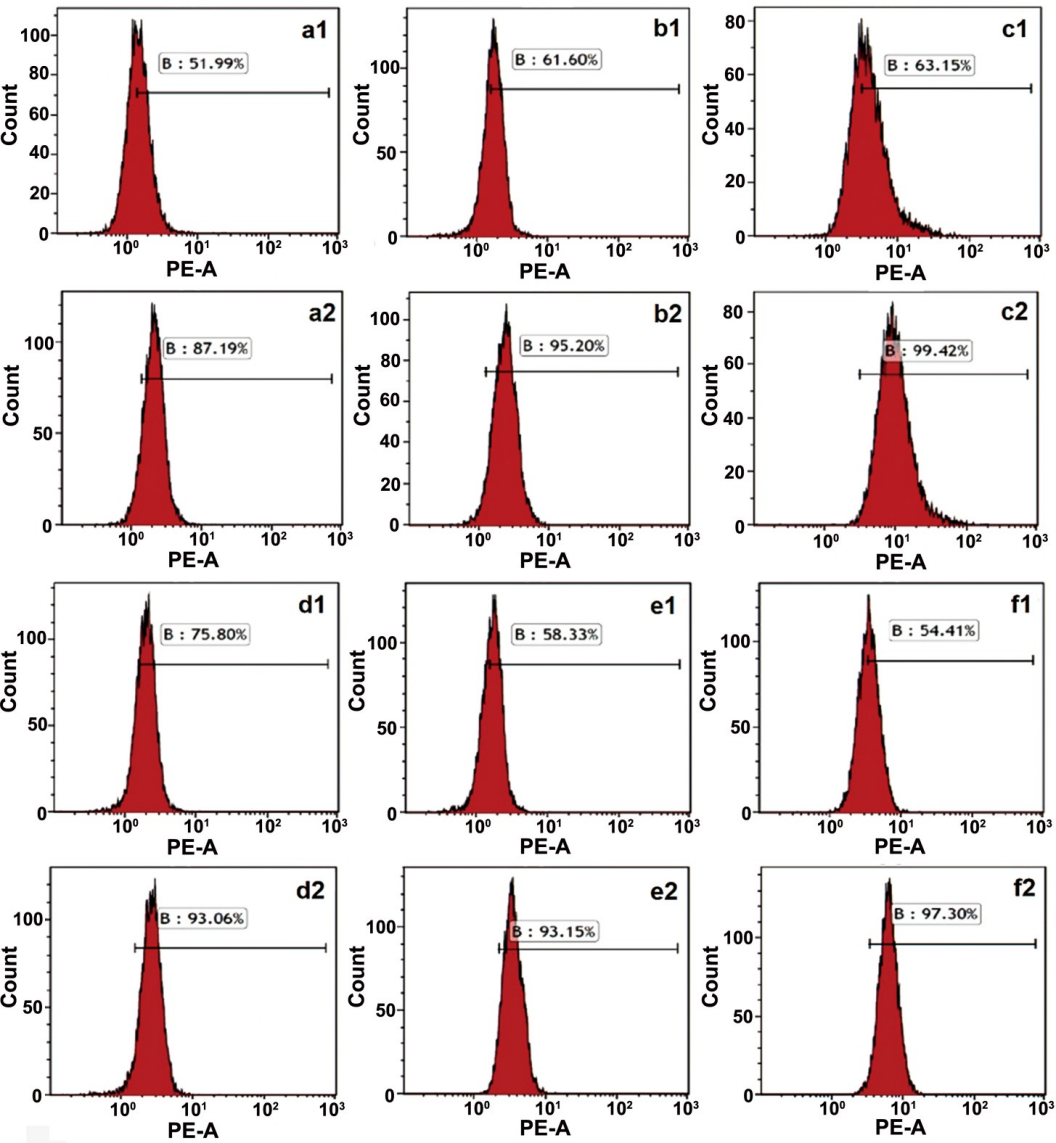
Intracellular uptake of doxorubicin measured by flow cytometry in Hela (a), HepG2 (b), MCF-7 (c), MDA-MB-231 (d), A549 (e) and NCI-H1299 (f) cells treated with DOX (1) and DOX-PEC-NC (2).

Similar as blank and doxorubicin loading PEC-NP [42] and liposome, the results of MTT assay showed that blank PEC-NC had no significant inhibitory effect (Fig 5A) and DOX-PEC-NC showed a time- and dose-dependent cytotoxicity in Hela, HepG2, MCF-7, MDA-MB-231, A549 and NCI-H1299 cells (Fig 5B), indicating the safety of blank drug delivery vehicle and effective anticancer activity of DOX-PEC-NC. Compared with DOX, DOX-LIP and DOX-PEC-NP [42], DOX-PEC-NC showed the highest inhibitory activity in the tested tumor cells at different concentration after 48- or 72-hour treatment, which had the lowest IC_50_ value (Table 3). The results measured by flow cytometry showed that DOX-PEC-NC enhanced the uptake of doxorubicin dramatically in the tested tumor cells (Fig 6), which may account for its stronger anticancer potency than doxorubicin solution. The same phenomena were also observed in the uptake test of DOX-PEC-NP [39] and DOX-LIP (Data was not listed.).

**Table 3:** IC_50_ of DOX, DOX-LIP and DOX-PEC-NC in cancer cell lines.

Cell strain	Time		IC_50_ (μg/mL)	
		DOX	DOX-LIP	DOX-PEC-NC
Hela	48 h	4.40	1.53	1.31
	72 h	2.37	1.05	0.54
HepG2	48 h	1.58	0.85	0.68
	72 h	0.76	0.49	0.33
MCF-7	48 h	1.57	0.58	0.46
	72 h	0.44	0.30	0.29
MDA-MB-231	48 h	1.27	0.62	0.45
	72 h	0.55	0.18	0.15
A549	48 h	1.17	0.69	0.53
	72 h	0.40	0.24	0.19
NCI-H1299	48 h	5.62	1.71	0.99
	72 h	1.33	0.87	0.54

#### MDR reversal action of DOX-PEC-NC

**Fig 7:**
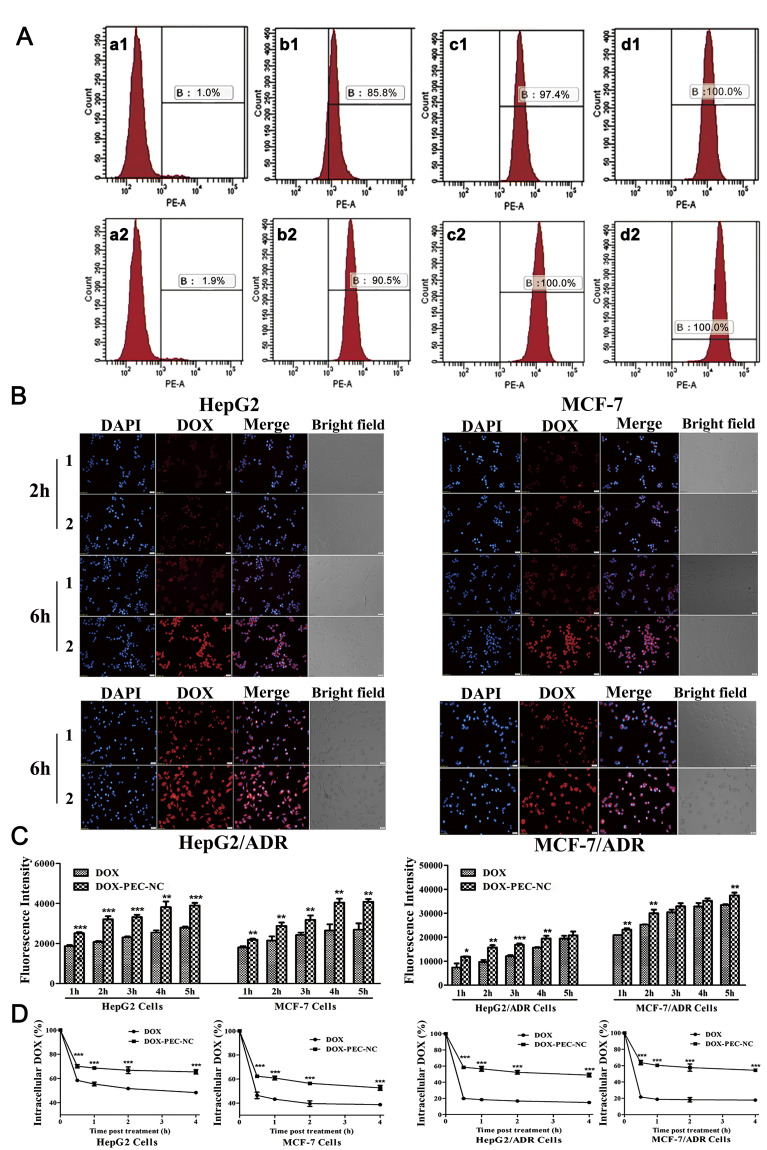
The reversal effect of DOX-PEC-NC on drug resistance in HepG2/ADR and MCF-7/ADR cells. The Fig A illustrated intracellular uptake of doxorubicin measured by flow cytometry in MCF-7/ADR cells treated with doxorubicin solution (1) and DOX-PEC-NC (2) at 0 (a), 5 (b), 10 (c) and 20 (d) μg/mL. The Fig B illustrated intracellular fluorescence intensity observed under inverted fluorescence microscope in HepG2, MCF-7, HepG2/ADR and MCF-7/ADR cells treated with doxorubicin solution (1) and DOX-PEC-NC (2) for 2 hours and/or 6 hours. The Figs C and D illustrated intracellular absorption and retention of doxorubicin measure by microplate reader in HepG2, MCF-7, HepG2/ADR and MCF-7/ADR cells treated with doxorubicin solution and DOX-PEC-NC (n = 3; *P < 0.05, **P < 0.01 and ***P < 0.001, compared with DOX).

As we known, MDR is a troublesome barrier of chemotherapy. We found the cytotoxicity and uptake of doxorubicin increased greatly in all the tested sensitive tumor cell lines after loaded in PEC-NC. May the PEC-NC affect the uptake and efflux of doxorubicin in drug resistant tumor cell lines to overcome MDR? Our results showed that the IC_50_ value of DOX-PEC-NC was much lower than that of doxorubicin solution in HepG2/ADR and MCF-7/ADR cells (Table 4), and it increased the uptake as well as decreased the efflux of doxorubicin in both sensitive and drug resistant tumor cell lines (Fig 7).

**Table 4:** Reversal effect of DOX-PEC-NC on drug resistance in HepG2/ADR and MCF-7/ADR cells.

Sample	IC_50_ (μg/mL)				Resistance fold		Reversal fold	
	HepG2	HepG2/ADR	MCF-7	MCF-7/ADR	HepG2/ADR	MCF-7/ADR	HepG2/ADR	MCF-7/ADR
DOX^a^	0.86	47.61	0.45	64.29	55.36	142.86	-	-
DOX-PEC-NP^a^	0.43	23.65	0.32	41.46	-	-	2.01	1.55
DOX^b^	0.76	48.25	0.44	63.53	63.49	144.39	-	-
DOX-LIP^b^	0.49	21.66	0.30	41.47	-	-	2.23	1.53
DOX-PEC-NC^b^	0.33	15.45	0.29	24.22	-	-	3.12	2.62

The superscript a and b meant IC_50_ values obtained from two batches of MTT assays, respectively.

As shown in Table 4, the IC_50_ value of doxorubicin solution in HepG2/ADR and MCF-7/ADR cells was much higher than that in HepG2 and MCF-7 cells, confirming the cells of obvious resistance to doxorubicin. Among DOX-PEC-NP, DOX-LIP and DOX-PEC-NC, DOX-PEC-NC significantly increased the sensitivity of drug resistant tumor cells and achieved the lowest IC_50_ value with the highest MDR reversal fold of 3.12 and 2.62 in HepG2/ADR and MCF-7/ADR cells, respectively. The intracellular fluorescence intensity measured by both flow cytometry and inverted fluorescence microscope demonstrated DOX-PEC-NC significantly strengthened the uptake of doxorubicin in HepG2/ADR and/or MCF-7/ADR cells (Fig 7A and 7B). It was found that the drug uptake increased with prolongation of incubation time from 2 hours to 6 hours in HepG2 and MCF-7 cells, which demonstrated more intensive fluorescence in cells after 6 hour incubation. DOX-PEC-NC treatment resulted in greatly stronger fluorescence than doxorubicin solution treatment in both sensitive and drug resistant HepG2 and MCF-7 cells (Fig 7B), indicating higher intracellular accumulation of doxorubicin caused by drug delivery of PEC-NC.

The results were further confirmed by intracellular drug absorption test, which showed that the absorption of doxorubicin in DOX-PEC-NC group was significantly higher than that in doxorubicin solution group in HepG2, MCF-7, HepG2/ADR and MCF-7/ADR cells (Fig 7C). The absorption of doxorubicin rose gradually with incubation time increased and tended to reach a plateau after 4 hour treatment. Possibly due to much higher concentration of doxorubicin used in drug resistant tumor cells, the influence of DOX-PEC-NC on drug absorption was weaker in HepG2/ADR and MCF-7/ADR cells than in HepG2 and MCF-7 cells. However, the increase of intracellular doxorubicin retention caused by DOX-PEC-NC was much higher in HepG2/ADR and MCF-7/ADR cells than in HepG2 and MCF-7 cells (Fig 7D). The difference of intracellular doxorubicin retention percentage between DOX-PEC-NC and doxorubicin solution group was about or less than 20% in HepG2 and MCF-7 cells, while about or slightly less than 40% in HepG2/ADR and MCF-7/ADR cells. It indicated that the efflux of doxorubicin was effectively reduced by drug delivery of PEC-NC.

It was reported that nanocarriers were internalized into cells via a non-specific endocytosis pathway and cross the cell membrane in an ‘invisible’ form, thereby preventing the drugs from being recognized by efflux pumps and resulting in a higher intracellular accumulation of the drug. The particles are internalized and release drugs near the peri-nuclear region away from membrane ABC transporters, thereby reversing the MDR of cancer cells to make them chemosensitive [43]. It was therefrom speculated that PEC-NC reversed the drug resistance of tumor cells by the same way of increasing uptake and decreasing efflux of chemotherapeutic agents.

### *In vivo* efficacy of DOX-PEC-NC in H_22_ tumor-bearing mice

**Fig 8:**
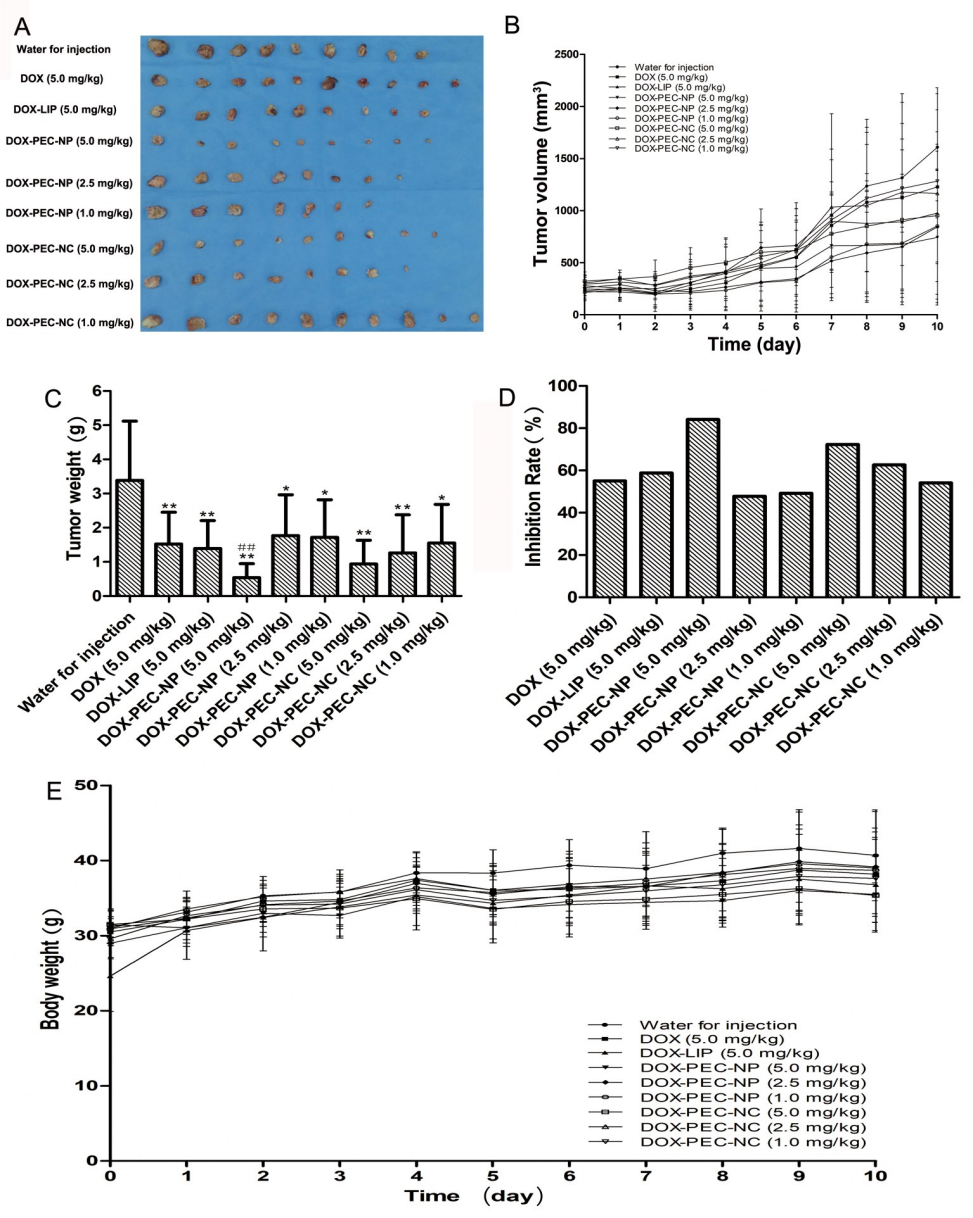
Antitumor efficacy of DOX-PEC-NC in mice bearing H_22_ tumor. Figs A, B and C were the photograph, volume, weight of tumor tissues of mice in each group after treatment, respectively. Fig D represented the inhibition rate of tumor growth in each group. Fig E was the body weight of mice in each group after treatment. Data were expressed as mean ± SD (n = 7–10; *p < 0.05 and **p < 0.01, compared with water for injection group; #*p* < 0.05 and ##*p* < 0.01, compared with doxorubicin solution group).

In view of the excellent *in vitro* anticancer activity of the developed doxorubicin loading pectin nanocell, we further evaluated its performance in mice bearing H_22_ tumor. With the treatment, the general status of animals including body weight (Fig 8E) showed no significant difference between groups, and only 11 mice were dead during experiment in model (1), DOX-LIP (1), DOX-PEC-NP (1, 2 and 3 in 5.0, 2.5 and 1.0 mg/kg group, respectively) and DOX-PEC-NC (1 and 2 in 5.0 and 2.5 mg/kg group, respectively) group with no abnormality observed by autopsy. Therefore, the number of mice of each group for data processing and results illustration in Fig 8 was 7 to 10.

As shown in Fig 8A–8D, DOX, DOX-LIP, DOX-PEC-NP and DOX-PEC-NC potentially inhibited the growth of tumor when compared with model group given water for injection (*P* < 0.05 or 0.01). With the increase of dose, the inhibition of DOX-PEC-NP and DOX-PEC-NC on tumor growth was enhanced (*P* < 0.01). Among the three different nanoscale drug delivery vehicles, at same dose of 5 mg/kg, DOX-LIP showed the weakest efficacy against tumor growth with an inhibition rate of 58.77%, which was close to the effect of doxorubicin solution (55.04%). The inhibition rate of DOX-PEC-NC and DOX-PEC-NP at dose of 5 mg/kg was 72.25% and 84.09%, respectively, which was much higher than that of DOX-LIP. DOX-PEC-NC showed dose-dependent increase of antitumor efficacy, and at dose of 1 mg/kg it exerted similar efficacy as doxorubicin solution (5 mg/kg) with an inhibition rate of 54.11%. Although the inhibition rate of DOX-PEC-NC at dose of 5 mg/kg was lower than that of DOX-PEC-NP, it achieved higher inhibition rate at dose of 2.5 and 1 mg/kg. In addition, at dose of 5 mg/kg, thymus index and spleen index of mice in doxorubicin group were significantly lower than those in model, DOX-PEC-NP and DOX-PEC-NC group (*P* < 0.05), and the indexes in DOX-PEC-NC group at dose of 2.5 and 1.0 mg/kg were close to those in model group (*P* > 0.05) (Table 5). The results indicated that DOX-PEC-NC greatly ameliorated the toxic side effect caused by doxorubicin.

**Table 5:** Thymus index and spleen index of H_22_ tumor-bearing mice in different groups ($\bar{X}\pm SD$, n = 7–10).

Group	DOX (mg/kg)	Thymus index	Spleen index
Water for injection	-	1.68 ± 0.74**	6.35 ± 1.19**
DOX solution	5.0	0.55 ± 0.38^##^	2.84 ± 1.66^#^
DOX-LIP	5.0	1.26 ± 0.96*	3.40 ± 1.44^#^
DOX-PEC-NP	5.0	1.01 ± 0.30*^;^^#^	5.51 ± 0.97**
DOX-PEC-NP	2.5	1.27 ± 0.80*	6.78 ± 3.17**
DOX-PEC-NP	1.0	1.41 ± 0.35**	6.60 ± 0.99**
DOX-PEC-NC	5.0	0.99 ± 0.43*^;^^#^	5.73 ± 1.34**
DOX-PEC-NC	2.5	1.43 ± 0.50**	6.17 ± 1.28**
DOX-PEC-NC	1.0	1.71 ± 0.33**	6.26 ± 1.01**

*p < 0.05 and **p < 0.01, compared with doxorubicin solution group

#*p* < 0.05 and ##*p* < 0.01, compared with water for injection group.

The DOX-PEC-NC likely achieved the better efficacy and lower toxic side effect through accumulation of nanoscale particles in tumor tissue due to the enhanced permeation and retention (EPR) effect [44], sustained release of drug and enhancement of intracellular uptake of drug. The DOX-PEC-NC accumulated at tumor site released doxorubicin slowly and produced lasting inhibition to tumor growth at not very high but effective concentration, which may lessen toxic side effect of doxorubicin as well.

## Conclusions

The chemotherapy resistance of tumor cells seriously reduces the therapeutic effect of chemotherapeutic agents. In this work, we developed DOX-PEC-NC of core-shell structure intending to improve anticancer efficacy and reverse MDR through nano-vehicle delivery of chemotherapeutic agents. The DOX-PEC-NC had better sustained release behavior than both doxorubicin loading liposome and nanoparticle. It demonstrated expected potency against tumor growth *in vitro* and *in vivo* by significantly enhancing intracellular accumulation of doxorubicin and prolonged drug release. DOX-PEC-NC was also found to reverse the drug resistance of tumor cells to a certain degree possibly due to increase of drug uptake and decrease of drug efflux. The slow release of drug enabled a lasting anticancer efficacy at a relatively low dose, thus enabled the reduction of toxic side effect of doxorubicin. The results herein indicated that PEC-NC would be a promising drug delivery vehicle for chemotherapeutic agents. Further studies are needed to confirm its reversal effect on tumor drug resistance *in vivo*, and targeting modification of PEC-NC may be an efficient way worthy of further attempt.

Also, we used sodium alginate as the material of nanoparticle core to prepare nanocells loading with cytarabine and doxorubicin in another study. The results showed that doxorubicin loading alginate nanocells had a drug loading rate close to that of DOX-PEC-NC, while cytarabine loading alginate nanocells demonstrated much lower entrapment efficiency. It indicated that different polysaccharide materials could be used to prepare nanoparticle core of nanocells and the solubility of drug might be an important factor affecting the drug loading capacity. Further investigation is expected to perform on more types of polysaccharide materials of nanoparticle core and different chemotherapeutic or other therapeutic agents to figure out the features of the novel nanovehicle, polysaccharide nanocells.
